# Cognitive decline in Parkinson’s disease: the complex picture

**DOI:** 10.1038/npjparkd.2016.18

**Published:** 2016-09-01

**Authors:** Roberta Biundo, Luca Weis, Angelo Antonini

**Affiliations:** 1 Parkinson’s Disease and Movement Disorder Department, “IRCCS, San Camillo” Rehabilitation Hospital, Venice-Lido, Italy

## Abstract

Mild cognitive impairment (PD-MCI) and dementia (PDD) are among the most frequent non-motor symptoms in Parkinson’s disease (PD). PD-MCI is six times more likely than age-matched controls to develop dementia and the PDD prevalence is 80% after 15–20 years of disease. Therefore, research has focused on the identification of early dementia biomarkers including specific cognitive at-risk profiles hoping to implement therapeutic interventions when they are most likely to be efficacious. However, given the heterogeneous neuropathological, neurochemical, and neuropsychological nature of cognitive deficits, definition of a comprehensive cognitive model of PDD is a challenge. Evidence from neuroimaging studies using different methods and techniques suggests that in addition to degeneration of the dopaminergic system, other mechanisms have a role including β-amyloid and tau deposition, and that specific cognitive scales could help identifying a malignant profile. Prospective studies combining neuroimaging techniques and specific cognitive tests are required to define the interplay between the various neurodegenerative processes and the contribution of structural disconnection in brain functional networks, heralding the development of dementia in PD.

## Introduction

Diagnosis of Parkinson’s disease (PD) relies on the presence of specific motor symptoms such as bradykinesia, resting tremor, rigidity, asymmetric manifestations, and good response to levodopa.^1^ PD patients also present several non-motor features often preceding motor signs by many years with a negative impact on quality of life, increased caregiver burden, and annual economic costs (estimated at ~14 billion in Europe).^2^ In particular, management of cognitive impairment is an area of growing clinical interest given its heterogeneous manifestations and the risk of dementia.^3^ Considering that up to 80% of PD patients after 15–20 years of disease have progressed to dementia,^4^ there is urgent need to identify early biomarkers to establish efficacious treatments and monitor cognitive deterioration. However, the presence of heterogeneous underlying pathophysiology and neuropsychological phenotype makes it difficult to define a unique model explaining the whole cognitive picture. Indeed, PD neuropathology demonstrates Lewy body-type degeneration in cortical and limbic structures,^5^ coexisting Alzheimer pathology (neurofibrillary tangle and senile plaques)^6^ as well as microvascular lesions with the involvement of subcortical structures.^7^ A post-mortem study showed that only 38% of PD patients with dementia (PDD) had “pure” or exclusive Lewy body pathology, whereas 59% presented combined Lewy body and β-amyloid plaques, and 3% concomitant Lewy body, β-amyloid plaques, and neurofibrillary tangles.^8,9^ Moreover, there is a significant positive relationship between cortical β-amyloid deposition and cognitive impairment,^10^ and β-amyloid accumulation is detected in one-third of non-demented PD.^11^ By contrast, only few neuropathology studies are available in Parkinson with mild cognitive impairment (PD-MCI) but results are heterogeneous.^12,13^

In addition, the coexistence of overlapping pathologies does not always match with the clinical manifestations due to the various cerebral distribution of abnormal protein deposits.^14^ This complex framework occurs in the context of multiple neurotransmitter deficiencies (including dopamine, noradrenaline, serotonin, and acetylcholine) and presence of genes that increase the dementia risk (such as ɑ-synuclein mutation (SNCA), the apolipoprotein ε4 (APOE4) allele, and the microtubule-associated protein tau (MAPT) H1 haplotype^7^). Finally, an approach based only on the clinical symptoms would be insufficient to define PD cognitive environment, given the poor sensitivity of the MCI diagnosis guidelines published in literature.^15^

This review outlines the most important issues related to the nature of the neuropsychological deficits, the identification of PD cognitive profile by a clinical semi-structured interview, and highlights the potential role of neuroimaging techniques such as magnetic resonance imaging, positron emission tomography, and single-photon emission computed tomography to increase the accuracy of early detection of cognitive dysfunction.

## Cognitive deficits in PD: the clinical neuropsychology-based approach

After an initial controversy about the presence of cognitive deficits,^16^ PD cognitive impairment and dementia are now well documented, and listed among the most prevalent and disabling non-motor symptoms.

Initially (1980–1990), cognitive deficits were considered executive in nature due to the dopamine depletion in the nigro-striatal pathways. This hypothesis was confirmed by several studies showing impairment in working memory, planning/sequencing (as in then Tower of London test), task-switching (as in the Wisconsin Card Sort test), response inhibition process (as in the Stroop task), memory recall, verbal fluency as well as many aspect of motor cognition (as psychomotor speed).^17^ They can be detected in early untreated patients, or even at premotor stage and worsen with disease progression.^18^ Moreover, evidence suggests spatiotemporal asymmetry of dopamine depletion at the striatal level characterized by an early involvement of the dorsolateral prefrontal circuit progressively extending to the orbito-frontal pathway involved in reward-based learning.^19^ This explains the non-linear effect on cognition of dopaminergic drugs. In line with the ‘dopamine overdose hypothesis’, dopaminergic medications may ameliorate dorsolateral prefrontal cortex-related executive functions, but overdose the intact portion with detrimental effect on orbito-frontal cortex-related executive functions such as decision-making and reversal learning tasks. In addition, as levodopa treatment only partially restores cognitive deficits,^20^ it is evident that a proportion of the impairment was not dopamine-related and neurotransmitter systems such as acetylcholine, noradrenaline, and serotonin are involved in cognition. More specifically, this regards specific attention problems (such as impaired vigilance with fluctuating level of alertness),^21^ memory deficits (with free recall retrieval more prominent at the earlier stage of the disease^22^ and an additional storage component memory present in PDD), and subtle visuo-spatial and perceptive impairments (difficulties with the perception of extra personal space^23^ and in recognizing objects based on their form).^24^ Unsolved questions regard the nature, dissociation, and progression of language and semantic impairments particularly syntactic, action-verb, and action semantic skills. Some authors posit that these deficits are epiphenomenal of executive dysfunction or impairment of selective attention or even working memory deficits,^25^ whereas others showed the presence of specific linguistic deficits (action-naming) without concurrent executive defects.^26^ Interestingly, a recent study found linguistic deficits in PD without MCI. In particular, action-naming and action-association were disrupted in the absence of executive deficits, suggesting a *sui generis* defect present since early disease stages.^27^

On the basis of the heterogeneity of cognitive deficits and cross-sectional and longitudinal evidence showing that visuo-spatial and semantic memory impairments are highly sensitive in detecting the transition to PDD,^28–30^ the ‘dual syndrome hypothesis’ was proposed. This hypothesis is based on the existence of two independent, partially overlapping syndromes in PD: (a) a frontal-striatal network dysfunction present at the early stage of the disease, which is dopamine modulated, leading to deficit in working memory, attention, planning, and response inhibition; (b) an additional more posterior cortical degeneration, which is associated with cholinergic loss and wherever present would lead to dementia.^31^ A major question regards the relationship between these two syndromes and more specifically on how many PD-MCI cases proceed to clinical dementia and what degree of overlap exists between these early deficits and the prevalence of dementia.

## How to cluster cognitive deficits

The concept of MCI has been applied in PD to improve early dementia detection and is used as an umbrella term to describe the heterogeneous deficits observed in the range between normal cognition and PDD.^15^ Prospective longitudinal studies showed that mild cognitive deficits can be detected in ~15–25% of newly diagnosed patients^28,32^ and could be present even before motor symptoms appear.^17,33^ PD-MCI patients have an annual rate of dementia between 9 and 15%,^34^ and clinical and demographic variables (mainly age, disease duration, and disease severity) affect the incidence.^35^ However, the low specificity of the MCI concept has contributed to this variability mainly because there are methodological differences in classification criteria (such as abnormal threshold range from −1 to −2 s.d.’s), there is no consensus about which and how many cognitive tests are needed for cognitive profiling and the cut-off scores are derived from inadequate normative data.^36–39^ For example, the threshold values indicated by the MCI Movement Disorder Task Force guideline do not place patient’s cognitive impairment in the continuum between PD without cognitive deficits and dementia, but can only be applied as dichotomous criteria for exclusion/inclusion in a given cognitive state. Varying those thresholds for the definition of PD-MCI affects frequency and clinical profile of MCI.^40^ Specific and more sensitive cut-off scores should be adopted for each test to increase accuracy of cognitive diagnosis. Our group previously observed that screening and diagnostic cut-off values for tests identified as good discriminator of PD-MCI would lie within the healthy subject range according to the published normative data, underling the risk of false-negative error in making MCI diagnosis.^41^ When Petersen^42^ and Winblad^43^ MCI subtype classification is applied to PD, it emerges that the non-amnestic single domain impairment predominates in PD-MCI^44^ and only few patients exhibit a more posterior cortical profile.^45^ Moreover, it is still uncertain which cognitive pattern best predicts the progression to PDD,^46,47^ although evidence suggests that impairment in semantic fluency and intersecting pentagon copying may be very sensitive.^35^ We recently assessed the discrimination power of several neuropsychological tests in differentiating cognitive stages in 105 PD patients (Parkinson without cognitive deficits versus PD-MCI versus PDD). We observed that attention/set-shifting, semantic memory, and language (category fluency task, naming test, prose memories, and similarities) as well as visuo-spatial functions (clock drawing test) were best in discriminating PD-MCI patients and an additional involvement of posterior cognitive functions (visuo-spatial, visuo-perceptive abilities together with language) in PDD,^28^ similar to that observed in Alzheimer disease.^48^

Overall, these findings, although heterogeneous, are in line with the presence of two cognitive profiles in PD-MCI associated with different aetiologies: (1) frontal-striatal-based executive–attention deficits in which the dopamine depletion has an important role; (2) posterior alterations^49^ associated with the increased risk of progression to PDD, presence of β-amyloid abnormalities, and further depletion of non-dopaminergic neurotransmitters. Finally, there is also evidence that PD cognition may show a non-linear decline depending on the impaired function.^50,51^

So far, longitudinal studies using PD-MCI published criteria demonstrated that their application may not be ideal in identifying the high dementia-risk PD cognitive profile likely because they do not take into account the contribution of single test deficits in predicting dementia.

## The neuroimaging evidence

Because of the aforementioned issues associated with the MCI concept in PD, almost 20 years neuroimaging studies based on PD-MCI/PDD published criteria, did not clarify the issue. Moreover, inclusion of small and poorly characterized subjects, heterogeneous methodological approaches (imaging preprocessing steps, motion artefacts, and limitation of specific techniques (voxel-based morphometry versus cortical thickness) that may be not high sensitive to detect cognitive decline at early PD stage) and medication status (on/off state) have increased variability in study findings.

Neuroimaging approaches have attempted to identify that brain components and correlated cognitive performance characterize the prodromal dementia profile (see Table 1 for a summary of the main studies) and discuss the contribution of anatomical ([gray (GM) and white matter (WM)]) abnormalities as markers of cognitive dysfunctions showing evidence of both frontal and posterior functional connectivity changes as possible biomarkers.

### Structural brain alterations

The PD-MCI structural phenotype emerging from studies using regions of interest analysis, voxel-based morphometry, and cortical thickness is heterogeneous.^52,53,55^ There is consensus on the presence of widespread cortical atrophy as well as increased and decreased thickness in neocortex and subcortical regions in PDD.^49,56^ Interestingly, impairment in specific domains correlates with both anterior and posterior cortical thinning in PD-MCI,^57^ underling the notion of widespread and shared brain network recruitment for high-level cognitive functions. Two longitudinal studies found a pattern of accelerated cortical thinning in PD non-demented compared with controls, in spite of similar controls cortical thickness pattern at baseline.^58,59^ However, these studies had limitations due to sample size^59^ and heterogeneous cohort.^58^ Conversely, a recent 2-year longitudinal study showed lower GM density at baseline in the prefrontal and insular cortex, and caudate nuclei in PD-MCI subjects who developed dementia.^54^ In the same line, 18 months’ longitudinal study showed bilateral temporal thinning at baseline in PD converters.^60^

Overall, these studies are in line with the dual syndrome hypothesis and support the concept of progressive temporo-parietal thinning together with frontal atrophy as a biomarker for progression towards dementia. It is still to be understood how the current PD-MCI categorization should be revised to achieve a finer delineation of the subgroups.

### White Matter alterations

Findings of WM microstructures across the full spectrum of cognitive status in PD are more consistent and a unique pattern of diffusivity changes underling impairment in distinct cognitive domains have been reported.^61,62^ Carlesimo *et al*.^66^ showed diffusion abnormalities correlating with memory deficits, in the posterior cingulated and hippocampus in non-demented PD, even in the absence of volume loss. Agosta *et al*.^64^ found structural WM alterations mainly located in the frontal regions in PD-MCI versus healthy controls, in spite of no GM atrophy. A recent study conducted in early PD showed increased regional mean diffusivity (MD) compared with healthy controls in frontal and parietal tracts correlating with semantic fluency and tower of London task, in the absence of reduction in both fractional anisotropy (FA) and GM volume.^65^ Indeed, increased MD, correlating with cognitive deficits, has been seen together with minimal FA alterations and absence of GM atrophy in both AD or PD.^63,76^ Taken together, these findings show that increased MD may be an early-stage marker of cognitive decline, and it could be detected before reductions of either FA and GM volume.

Given these consistent WM findings, some authors posit that PD could be primarily the result of synaptic dysfunctions (due to the presence of Lewy neurites in the axon), which subsequently lead to cell death.^11,77^ This evidence underlies the potential role of DTI as preclinical dementia biomarker in the absence of atrophic changes.

### Resting-state and functional connectivity studies

In the context of an integrated model of brain function, neuroimaging studies in healthy subjects showed that coherent and reliable pattern of spontaneous neuronal oscillation are observed at rest.^78^ Analyzing the correlated and anti-correlated regions activity, it has been observed that cognitive processing is subserved by a large-scale intrinsic connectivity networks (ICNs).^79^ In particular, the default mode network (DMN),^80^ the dorsal–ventral attention network (DAN),^81^ the fronto-parietal network (FPN),^82^ the central–executive network (CEN),^83^ the visuo-spatial, and the salience network (SN) has been extensively investigated.^67^ The study of these ICNs suggested that task-free or resting-state techniques are a useful tool to better understand human behavior and diseases.^84^

Previous studies suggested that resting-state brain organization could provide insight on how the brain reacts during external tasks.^85^ For example, dysfunctional connectivity among the SN, CEN, and DMN has been in several brain disorders,^86^ including PD.^73,87^ In particular, Putcha *et al.*^87^ found an aberrant positive coupling between DMN and right CEN, compared with the expected anti-correlation seen in HC,^88^ possibly reflecting a failure to suppress DMN activity or to modulate top-down signals between DMN and CEN.^89^ They also found a reduction in functional coupling between SN and right CEN in PD when compared with HC. Insula and dorsal anterior cingulated cortex, key nodes of the SN,^83^ are anatomically and functionally connected with CEN.^90^ Interestingly, network changes may reflect an increased PD-related pathological burden in the striatum and insula as suggested by Christopher *et al.*,^74^ who found that combined striatal-insula dopamine depletion predicted executive dysfunction in PD-MCI. The role of the fronto-insular cortex has been recently recognized by the finding of reduced functional connectivity in right fronto-insular regions and the DAN (which was correlated with attention and executive network) in treated PD-MCI.^68^ Combined with evidence that dopamine modulates resting-state patterns of coupling between DMN–FPN–DAN networks,^91^ it can be inferred that ICNs changes mediated by insular dopaminergic denervation have a role in dopamine modulated fronto-striatal deficits.^92^ These results, corroborate the presence of a frontal syndrome underlying the association between ICNs changes and dopamine-related cognitive deficits in PD.^69^ However, other studies observed the presence of posterior functional connectivity changes in PD.^72^ Tessitore *et al*.^70^ reported decreased functional connectivity of the right medial temporal lobe which, projecting to the hippocampus, predicted memory performance, and decreased inferior parietal cortex, which predicted visual-spatial scores, in cognitively unimpaired PD patients on medication. Baggio *et al.*^68^ observed increased connectivity between DMN and posterior cortical regions that were also atrophic. Moreover, ICN changes were associated with visual-perceptual deficits. A longitudinal study,^71^ using a synchronization likelihood as a measure of coupling, observed resting-state functional connectivity reductions mainly involving posterior cortical regions in PD-MCI on therapy. Interestingly, those functional connectivity changes correlated with cognitive decline. Other studies reported greater cholinergic denervation in PD with dementia compared with PD^75^ and the synergic role of acetylcholine and dopamine activity in predicting cognitive performance.^93^ In summary, current evidence on functional connectivity highlights that both frontal and posterior syndromes have resting-state correlates, with posterior alterations detectable also at preclinical cognitive stage and most likely predictor of worse cognitive outcome. Indeed, fronto-striatal executive deficits are mainly mediated by dopaminergic alterations, whereas additional neurotransmitters depletion, including cholinergic deficits, are associated with widespread cortical atrophy.^31,45^

## Discussion and Conclusions

Dementia in PD causes significant disability and leads to institutionalization and death. Early identification of patients at risk for early dementia is required to properly apply advanced treatments such as deep brain stimulation and design, and implement therapeutic interventions early when they are most likely to have efficacy. In the present review, we have shown the complexity in providing a comprehensive cognitive model of PDD, given the neuropathological, neurochemical, and neuropsychological heterogeneity of cognitive profiles. Loss of nigro-striatal dopamine neurons, cholinergic projections, limbic, and cortical alpha-synuclein positive Lewy bodies are associated with motor and cognitive deterioration.^5,11^ Although the presence of two coexisting cognitive profiles in PD is consistently reported across studies, the aforementioned evidence also suggests that each clinical, neuropsychological, and magnetic resonance imaging assessments cannot by themselves reliably discriminate PD patients who are likely to progress to dementia. Moreover, the presence of posterior functional connectivity abnormalities in cognitively unimpaired patients^70^ casts doubts about the temporal aspect of the dual syndrome hypothesis, which would postulate initial frontal–executive abnormalities followed by a subsequent additional posterior deficits. The confounding factors could be the *a priori* cognitive categorizations in PD-MCI and PDD, adopting heterogeneous approaches to classify patients without taking into account the temporal onset of cognitive features in respect of motor symptoms. Indeed, defining the timing of cognitive decline in PD is important and needs to be clarified. Intriguingly, neuropathology studies give us an idea of the importance of defining the onset of cognitive deficits. PD, PDD, and dementia with Lewy bodies (DLB) patients share the presence of α-synuclein aggregates in Lewy bodies and neuritis, and different timing in the onset of cognitive and motor manifestations may reflect the diverse regional burden and cerebral distribution of the pathology. Moreover, β-amyloid deposition is a frequent feature of DLB strongly affecting clinical manifestations.^94^ In PDD, duration of parkinsonism before dementia is associated with different patterns of brain pathology and neurochemical abnormalities.^95^ Similarly, retrospective data from the UK Brain Bank demonstrated that very long disease duration (up to 28 years) showed no clinical dementia at death irrespective of the presence of cortical Lewy bodies similar to that observed in DLB.^9^

In conclusion, a single model for Lewy body cognitive abnormalities and dementia should be adopted. In particular, prospective studies combining neuroimaging and sensitive cognitive tests should clarify the interplay between the various neurodegenerative processes, and the contribution of structural disconnections in brain functional networks, heralding the development of dementia. We suggest that additional pathology has a role in the early development of dementia and this includes β-amyloid deposition possibly in specific brain regions. Finally, the mini-mental state examination, a general cognitive scale widely used in AD, which is relatively insensitive in detecting cognitive abnormalities in the early phases of PD, but it shows a rapid decline (yearly point loss of 7–8%) in the presence of dementia,^28,96^ may be a valuable cognitive biomarker to identify patients at risk for dementia.

## Figures and Tables

**Table 1:** Main imaging studies in PD patients with cognitive impairment and/or dementia screened using recent consensus criteria

*Methods*	*Study group*	*Participants*	*Study design*	*Brain alterations*
VBM	Weintraub *et al.*^52^	60 PD non-demented	Longitudinal 2-year FU	PD-MCI: hippocampus and temporal lobe GM atrophy.
	Melzer *et al.*^53^	57 PD-CNT, 23 PD-MCI,16 PDD, 34 HC	Cctional	PD-MCI: temporal, parietal, frontal cortex, amigdala, right putamen, and hippocampal GM atrophy. PDD: additional medial temporal lobe, lingual gyrus, posterior cingulate gyrus, and bilateral caudate atrophy.
	Lee *et al.*^54^	51 PD-MCI, 15 converters/36 non-converters	Longitudinal 2-year FU	PD-MCI converters showed lower GM density in the left prefrontal areas, left insular cortex, and bilateral caudate nucleus compared with non-converters.
VBM and TBSS and 23I-IMP SPECT perfusion	Hattori *et al.*^55^	32 PD-NC, 28 PD-MCI, 25 PDD, 29 DLB, 40 HC	Cross-sectional	PDD: more cerebellum, bilateral thalami, insular cortex, and the parietal and occipital cortex atrophy. Bilateral parietal tracts atrophy correlates with lower mini-mental state examination scores; PDD occipital and parietal hypoperfusion. No GM difference between PDD and DLB.
Cortical thickness	Biundo *et al.*^49^	15 PD-CNT 14 PD-MCI, 21 HC	Cross-sectional	PD-MCI: thinning in right supramarginal, DLPFC, orbito-frontal, fusiform, hippocampus, superior parietal, and cuneus. Thickening in left occipito-parietal and superior/inferior temporal areas.
	Pagonabarraga *et al.*^56^	26 PD-NC, 26 PD-MCI, 20 PDD, 18 HC	Cross-sectional	Progressive cortical thinning from PD-MCI to PDD in enthorinal cortex, anterior temporal pole, parahippocampal, fusiform gyrus, bankst, lingual gyrus, cuneus, and precuneus.
	Pereira *et al.*^57^	90 PD-NC, 33 PD-MCI, 56 HC	Cross-sectional	PD-MCI: thinning in left precuneus with a progressive involvement in inferior temporal precentral, superior parietal, and lingual areas.
	Hanganu *et al.*^58^	15 PD-CNT, 17 PD-MCI,18 HC	Longitudinal 1- and 5-year FU	PD-MCI: thinning in temporal and medial occipital lobe, and subcortical volume reduction in nucleus accumbens and amygdala correlating with a rate of decline.
	Ibarretxe-Bilbao *et al.*^59^	16 early PD, 15 HC	Longitudinal 3-year FU	PD patient showed cortical thinning in bilateral fronto-temporal regions and amygdala volume reduction.
	Mak *et al.*^60^	66 PD-CNT, 39 PD-MCI, 37 HC	Longitudinal 1- and 5-year FU	PD-MCI converters at FU showed bilateral cortex thinning at baseline.
	Lee *et al.*^54^	51 PD-MCI, 15 converters/36 non-converters	Longitudinal 2-year FU	PD-MCI converters showed lower GM density in the left prefrontal areas, left insular cortex, and bilateral caudate nucleus compared with non-converters.
ROI-based DTI parcellation	Theilmann *et al.*^61^	25 PD non-demented, 26HC	Cross-sectional	WM anterior corona radiata and caudal anterior cingulate atrophy correlate with worse cognitive performance.
	Zheng *et al.*^62^	16 PD	Cross-sectional	Anterior limb of the internal capsula, genu of the corpus callosum, fornix, and cingulum atrophy correlate with cognitive decline and specific patterns are associated with different cognitive domains.
TBSS	Melzer *et al.*^63^	63 PD-CNT, 28 PD-MCI, 18 PDD, 32 HC	Cross-sectional	WM progressive deterioration (lower FA and higher MD) correlate with cognitive performance worsening.
TBSS and VBM	Agosta *et al.*^64^	10 PD-CNT, 33 PD-MCI, 33 HC	Cross-sectional	WM atrophy in anterior part of corona radiata, genu, and body of corpus callosum, anterior inferior fronto-occipital, uncinate, and superior longitudinal fasciculi, bilaterally (FA,MD) in PD-MCI, in the absence of GM atrophy.
TBSS and VBM	Duncan *et al.*^65^	125 PD, 50 HC	Cross-sectional	WM atrophy in cingulum, superior longitudinal fasciculus, inferior longitudinal fasciculus, and inferior fronto-occipital fasciculus in PD patient with language and executive problems. No GM atrophy.
	Carlesimo *et al.*^66^	25 PD non-demented, 25 HC	Cross-sectional	High hippocampal MD value, without volume change, correlates with worse memory performance.
Resting-state fMRI	Gorges *et al.*^67^	14 PD-CNT,17 PD-CI, 22 HC	Cross-sectional	Default mode frontal region hyper connectivity in early-stage cognitive preserved PD and hypoconnectivity with cognitive impairment in the same regions.
	Baggio *et al.*^68^	32 PD-CNT 23 PD-MCI, 36 HC	Cross-sectional	PD-MCI showed reduced connectivity between the dorsal attention network and right fronto-insular regions, associated with worse performance in attention/executive functions. The default mode network displayed increased connectivity with medial and lateral occipito-parietal regions, associated with worse visuo-spatial performance, and with occipital-parietal atrophy.
	Amboni *et al.*^69^	21 PD-CNT, 21 PD-MCI, 20 HC	Cross-sectional	PD-MCI patients, but not PD-CNT, compared with healthy controls, showed decreased functional connectivity in bilateral prefrontal cortex within the fronto-parietal network. No GM abnormalities in PD-MCI versus PD-CNT.
	Tessitore *et al.*^70^	16 PD-CNT, 16 HC	Cross-sectional	PD-CNT patients showed decreased DMN connectivity that significantly correlated with cognitive parameters but not with disease duration, motor impairment, or levodopa therapy.
	Olde Dubbelink *et al.*^71^	36PD, 12 HC at FU	Longitudinal 3-year FU	Age-independent functional connectivity changes were most prominent for posterior parts of the brain and correlated across time with cognitive decline.
	Chen *et al.*^72^	19 PD-CNT, 11 PDD, 21 HC	Cross-sectional	Cognitive decline in PD is associated with functional connectivity damage of posterior cingulate-right middle temporal lobe and microstructural damage of left hippocampus.
[(123)I][β]-CIT PET	Ravina *et al.*^73^	491 PD	Longitudinal 2-year FU	Reduction of dopamine binding after 2 years do not correlate with cognitive decline.
DTBZ/(11)C-FLB 457 PET	Christopher *et al.*^74^	11 PD-CNT, 11 PD-MCI, 11 HC	Cross-sectional	Striatal dopamine depletion in the associative (i.e., cognitive) subdivision as well as reduced D2 receptor availability in the bilateral insula in PD-MCI compared with PD-CNT and HC.
(FDOPA), (MP4A), (FDG)-PET	Klein *et al.*^75^	9 PD non-demented, 8 PDD, 6 DLB	Cross-sectional	Reduced FDOPA uptake in the striatum and in limbic, and associative prefrontal areas in all patient groups. PDD and DLB showed same hypomethabolism (FDG)- and cholinergic (MP4A)-binding reduction in the neocortex with increasing signal diminution from frontal to occipital regions. Patients with PD without dementia had a mild cholinergic deficit and no FDG reductions versus controls.

Abbreviations: AD, Alzheimer’s disease patients; DLB, dementia with Lewy body patients; DMN, default mode network; DTI, diffusion tensor imaging; DLPFC, dorsolateral prefrontal cortex; DTBZ, (11)C-dihydrotetrabenazine; FA, fractional anisotropy; FDG, (18)fluorodeoxyglucose; FDOPA, (18)fluorodopa; fMRI: functional magnetic resonance imaging; FU, follow-up; GM: cerebral gray matter; HC, healthy controls; MD, mean diffusivity; MP4A, *N*-(11)C-methyl-4-piperidyl acetate; PDD, Parkinson’s disease patients with dementia; PD-MCI, Parkinson’s disease patients with mild cognitive impairment; PD-CNT, Parkinson’s disease patients without cognitive impairment; PET, positron emission tomography; ROI, region of interest; SPECT, single-photon emission computed tomography; TBSS, tract-based spatial statistics; VBM, voxel-based morphometry; WM, cerebral white matter.
